# Status, distribution and habitat use by Mugger crocodile (*Crocodylus palustris)* in and around Koshi Tappu Wildlife Reserve, Nepal

**DOI:** 10.1016/j.heliyon.2022.e10235

**Published:** 2022-08-17

**Authors:** Divya Bhattarai, Saurav Lamichhane, Pratik Pandeya, Santosh Bhattarai, Jeetendra Gautam, Ram Chandra Kandel, Chiranjibi Prasad Pokheral

**Affiliations:** a Agriculture and Forestry University, Makwanpur, Hetauda, Nepal; b Nepal Conservation and Research Center, Ratanagar-06, Chitwan, Sauraha 44204, Nepal; c IUCN-SSC-Crocodile Specialist Group, Australia; d Department of National Parks and Wildlife Conservation, Babarmahal, Kathmandu, Nepal; e National Trust for Nature Conservation, Khumaltar, Lalitpur, Nepal

**Keywords:** Habitat survey, Transects, Variables, Generalized linear model, Binary logistic regression

## Abstract

Mugger crocodile is found in various locations of Nepal, ranging from Eastern to Western lowlands, and is also a resident crocodilian of the Koshi Tappu Wildlife Reserve (KTWR). Fewer studies have been carried out on the status and distribution of this crocodilian with limited information on its habitat characteristics. This study sets forth to highlight the population status, distribution and habitat use by mugger crocodile in and around KTWR. Detailed surveys were conducted in the rivers, natural and private ponds of the KTWR in December 2020. Every 500 m along the survey transects, habitat characteristics were recorded in each habitat station as part of the habitat survey. The dependent variables were presence or absence of mugger crocodile in each habitat station. Meanwhile, the independent variables included habitat predictors such as; water depth, slope, elevation, distance to roads, distance to settlements, bank substrate, and human disturbance. Generalized Linear Model under binary logistic regression was employed to test variables associated with presence and absence of muggers for statistical significance. The population survey of the muggers was assessed by day counts either using dugout canoe or walking along transects. During the research period, 35 individuals of muggers were recorded. The probability of mugger sighting augmented with increased slope and depth. 34 muggers (97.14 %) were basking, while 1 (2.85%) were seen swimming out of the total muggers detected. In private and public ponds, 22 muggers were sighted, while 13 were sighted in the main Koshi river, its branches, and the Moriya river. Sixteen muggers (45.71 %) were sighted within the reserve, while the remaining 19 muggers (54.28 %) were sighted in the buffer-zone areas. The probability of mugger sighting varied considerably with regard to slope and water depth among the seven habitat predictors examined. In lieu of prevailing fishing pressure in the river systems of the KTWR and easy availability of prey species for muggers in private fishponds; the muggers are likely use private fishponds more frequently. In order to subdue the muggers inside the KTWR, the reserve authority has a vital role for proper habitat management decisions and regulation of fishing activities.

## 1 Introduction

Of the 24 species of extant crocodylians globally only two are found in Nepal, the Gharial (*Gavialis gangeticus*) and the Mugger (*Crocodylus palustris*) (Lamichhane et al., 2021), which are sometimes sympatric (Groombridge, 1982; De Silva and Lenin, 2010). Muggers occur in India, Pakistan, Nepal, and Iran, with status varying between range states (De Silva and Lenin, 2010); with global IUCN Red List status as Vulnerable (Choudhary and De Silva, 2013), They are enlisted on Appendix I of the Convention on International Trade in Endangered Species of Wild Fauna and Flora since 1975.

In Nepal, muggers are found throughout lowlands where suitable habitat is protected, illegal hunting and other anthropogenic disturbance are minimal (Shah and Tiwari, 2004). However, due to habitat modification and disturbance; the muggers are locally extirpated from many wetland and rivers in Nepal and now are restricted as isolated populations, mainly in protected areas such as ShuklaPhanta, and Bardia National Parks and Ghoda-Ghodi lake complex in the Western lowland; Chitwan National Park in the central lowland and Koshi Tappu Wildlife Reserve in the Eastern lowland of Nepal (Whitaker and Andrews, 2003; Lamichhane et al., 2021). Some studies on mugger crocodile population estimation have been conducted by earlier researchers. Maskey and Schleich (1992) and Andrews and McEachern (1994) estimated 107–148 and 200 muggers in Nepal respectively. Likewise Khadka et al. (2014) and Lamichhane et al. (2021); recorded 245 muggers in Chitwan National Park and 26 muggers in Ghodaghodi lake complex respectively. The countrywide population of mugger is not available, however it is predicted that the total population of muggers in Nepal are between 400 and 500 (Baral and Shah, 2013).

Schleich et al. (2002) mentioned the occurrence of 10–12 muggers in the Koshi River. Similarly, based on direct observation, Goit and Basnet (2011) reported 21 and Baral and Shah (2013) reported 16 muggers in KTWR. Overgrazing and the movement of livestock along shorelines contribute to soil erosion which leads to loss of suitable habitats for crocodiles (Goit and Basnet, 2011). Excessive use of fishnets and overexploitation of fishes are also detrimental to crocodiles (especially juvenile muggers in KTWR) as they become entangled in nets and are either drowned or killed by fishermen (Maskey, 2008; Goit and Basnet, 2011; Baral and Shah, 2013). The collection of eggs and slaughtering of crocodiles for meat and body parts has caused population decline and disparate sex ratio (Maskey, 2008). Most crocodilian studies in Nepal are limited to Chitwan and Bardia National Parks largely focusing on gharial crocodile and its population status and conservation breeding. Past studies on muggers have provided limited information on habitat characteristics of the species (Lamichhane et al., 2021). Therefore, this study sets forth to highlight the existing knowledge gap, provide information on habitat use, current population status of mugger crocodile in the KTWR with an aim to generate updated information on the species to inform effective conservation planning and management decisions.

### 1.1 Study area

The research was carried out in the Koshi Tappu Wildlife Reserve (KTWR) and its surrounding buffer zone areas. The KTWR is a category IV protected area that was established in July 1976 and encompasses an area of 176 km^2^ (KTWR, 2018). It is located in the districts of Sunsari, Saptari, and Udayapur in the south-eastern Terai of Nepal. The geographic location of the reserve ranges from 26º34′ to 26º45′N and 86º55′ to 87º05′E with an altitude of 80–95 masl. The bioclimatic zone of the reserve is tropical with an annual rainfall of 2019 mm, and the rainy season falls within a discrete wet season from June to September (Limbu and Subba, 2011). The maximum and minimum temperatures are 38 °C and 8 °C respectively (KTWR, 2020). It has been enlisted as a Ramsar site of international importance since 1987. The reserve hosts the only viable population of Wild water buffalo (*Bubalus arnee*) in Nepal and several globally threatened aquatic fauna including Gangetic dolphin (*Platanista gangetica*) and smooth coated otter (*Lutrogale perspicillata*) (Chettri et al., 2013). The reserve is recognized as an Important Bird Areas of Nepal with home to endangered bird species (Baral and Inskipp, 2005).

**Figure 1:**
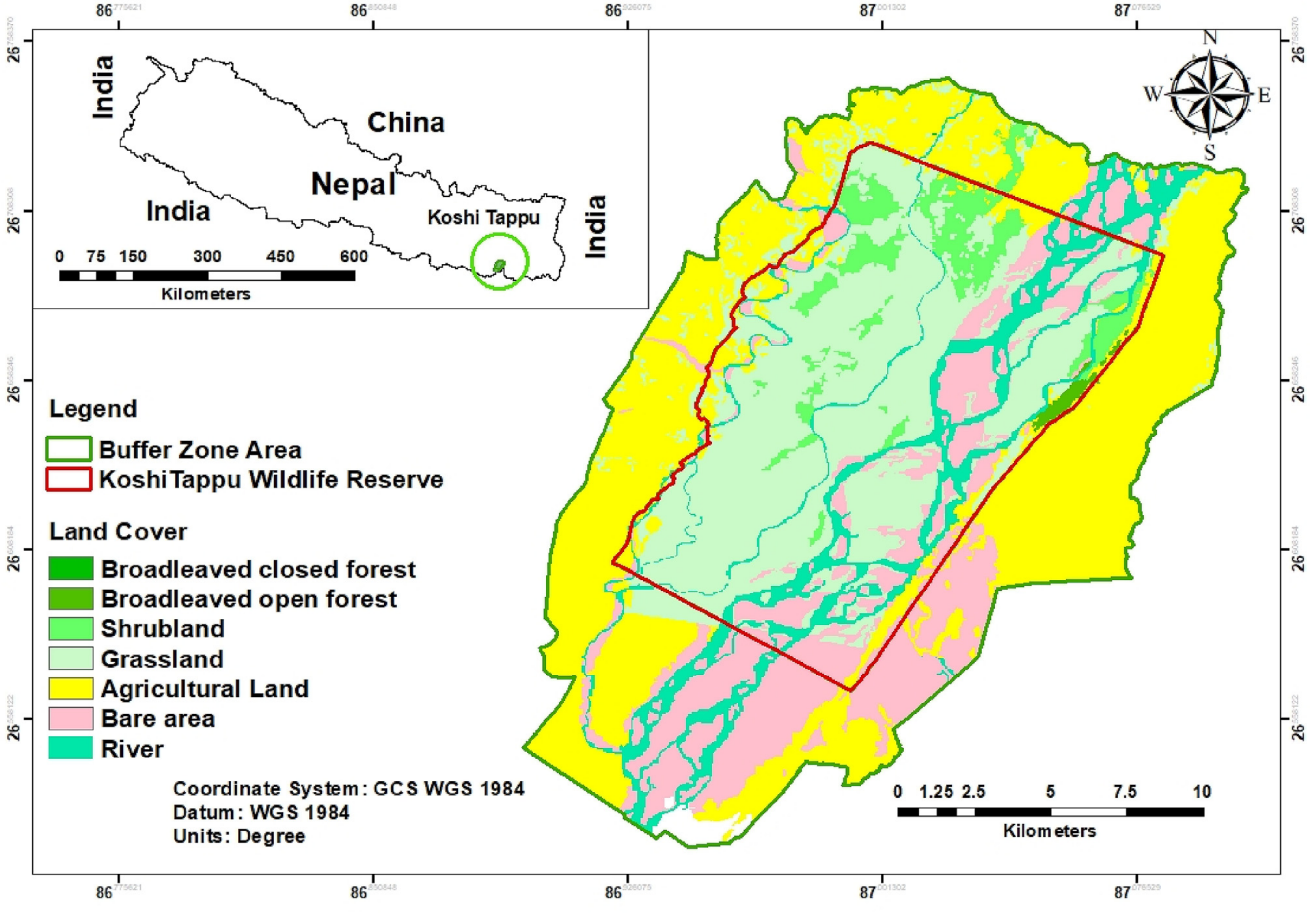
Location map of the study area.

The buffer zone around the reserve was established in 2004 by the Nepal government to involve the local community in participatory conservation and benefit-sharing. Presently, buffer-zone covers 4 municipalities and 1 rural municipality. Total 14.685 households with population of 84,423 people are residing in the buffer zone (KTWR, 2018). In the eastern buffer zone, seepage of water from the Koshi river has created a biodiversity rich swampy area which is lately being converted into series of private fishponds (Mishra et al., 2020). The wetlands of the reserve have also been regarded as an important area that provides a range of goods and services (Sah, 1997). The residents of the reserve have been dependent on natural resources of the reserve for decades; the fishery of the area is known to support the livelihoods of many households of the buffer zone (Chaudhary et al., 2016) (see Figure 1).

## 2 Methodology

A preliminary survey was conducted in and around KTWR from 20 to 30th November, 2020 to explore the potential presence sites of the mugger crocodiles. During the survey, informal interviews were conducted with key informants, park members, field guides and Buffer Zone User Committee (BZUC) to obtain information on the mugger presence in the area. Our team conducted field visits prioritizing fixing of transects for the detailed survey.

### 2.1 Population status survey

Based on information obtained from preliminary survey, population status of mugger was conducted in five transects from 02 to 16 December 2020 from 09:00 to 17:00 h. The survey was carried out during daytime in the winter season to maximize the probability of sighting coinciding the basking time of the muggers (Lamichhane et al., 2021; Neupane et al., 2020; Acharya et al., 2017). Crocodile surveys in Nepal are conducted during day light hours in the winter season between October and February (see Acharya et al., 2017; Lamichhane et al., 2021). During the winter, all individuals come out of water for basking and thermoregulate on the banks. The basking individuals in each segment were counted, and photographed. The little vegetation size class on the banks in basking sites did not create size-class related visibility bias.

The sightings were all sequential, and no individual crocodile was duplicated during the surveys. The survey was conducted with team of five members including boatmen. Traditional dugout canoe was used rather using motor engine boat to avoid disturbance to the mugger and to increase the probability of sighting. The dugout canoe was used in the main river (transect I) and remaining transects that could be assessed by walking were surveyed on foot. Transect refers to stretches (Goit and Basnet, 2011) along which the survey was carried. Both river banks were sampled for the presence of the mugger crocodile. The Total Length (TL) of muggers sighted were estimated visually to categorize them into various age classes as hatchlings (*<*30 cm); yearlings (30–50 cm); juveniles (50–125 cm); sub-adults (125–180 cm); and adults (*>*180 cm) following Khadka et al. (2014). Binoculars (Vortex optics 8 × 42) and Olympous (10 × 50) were used for observation.

**Transect I**: It included the main Koshi river where water was deep and fast and extended from Prakashpur to Haripur and covering 13.5 km. The dominant vegetations along the bank of this transect were *Saccharum spontaneum*, and *Vetiveria zizanioides.*
**Transect II**: This transect was in the Eastern side of the reserve, and had a length of 13 km. It included the Gohi tal area, eastern branch of the Koshi river and seepages made by the Koshi river. The dominant vegetation in this transect were *Dalbergia sissoo*, *Bombax ceiba* and *Typha elephantina.*
**Transect III**: This transect included chain of private ponds in the buffer zone area from Prakashpur to Shreepur in the Eastern side of the reserve. The major vegetations included *Typha elephantina* and *Eichhornia crassipes.* The length of this transect was 11 km.
**Transect IV**: It included natural pond and small river tributary from Bhagalpur to Badgamma with a length of 12 km. The major areas were Kamal Daha pond, Gheguwa lake and Moriya river. This area included mainly open grasslands.
**Transect V**: This transect included the Western branch of Koshi river. The length of this transect was 10.5 km.

### 2.2 Habitat survey

For habitat survey, we divided the length of each transect into 500 m segment following Lamichhane et al. (2021) and Neupane et al. (2020). Thus we generated total 120 stations for the study. However, in few stations we only noticed landmass and forest areas with the complete absence of water bodies in them. Therefore, to generate habitat similarities between all the stations, we removed those stations and finalized 110 stations for the final habitat modeling (see Table 1).

**Table 1:** Number of transects along with their major features.

S.N	Transects	Major features included	Total length (km)	Number of stations
1.	I	Main Koshi river	13.5	27
2.	II	Gohi tal, natural seepages made by the Koshi river	13	26
3.	III	Chain of private ponds	11	22
4.	IV	Kamal Daha pond, Gheguwa lake, Moriya river	12	24
5.	V	Western branch of Koshi river	10.5	21

The presence/absence of the mugger crocodile was determined through direct observation. The GPS location of each place where the muggers were observed was recorded. We fixed seven habitat predictors (water depth, slope, elevation, distance to roads, distance to settlements, bank substrate, and human disturbance) as independent variables for the study. The water depth of the Koshi river, Moriya river, natural ponds and community pond was measured with long graduated bamboo pole. Similarly, water depth of the private ponds was inquired with the pond owners. The slope and elevation of each station was recorded using clinometers and GPS respectively. In case of the slope, slope of both banks of the river were measured and averaged to find the final slope.

Shapefile of road and settlement of Nepal were extracted from OCHA Nepal (https://bit.ly/3T9OuSC) and polygon was made covering the study area. We calculated the distance of the roads and settlements from each station using the Euclidean distance tool in ArcGIS10.5 (ESRI, 2017). The bank substrate and human disturbance in each station were recorded based on direct observation. We divided the bank substrate into six classes (grassy bank, muddy and grassy bank, muddy bank, sandy and grassy bank, sandy and muddy bank, sandy bank) along the survey transects. The bank was taken as grassy bank if it was densely covered with varieties of vegetation such as *Saccharum* sps., *Cyperus* sps., *Eleusine indica, Imperata cylindrica etc.*; muddy and grassy bank if covered with fine clay with vegetation; muddy bank if covered with fine clay without vegetation; sandy and grassy bank if was the combination of sand with vegetation; sandy and muddy bank if was the combination of sand and clay with absence of vegetation; sandy bank if covered with fine sand without vegetation. Human disturbances were recorded on “Yes” or “No” basis for each station (Lamichhane et al., 2021). If we observed fishing activities, human disposed materials (fishing net, plastics), livestock grazing etc. in particular station then we recorded that station as disturbed one (coded 1), and if not, then non-disturbed one (coded 0) (see Table 2).

**Table 2:** Variables used in binary logistic regression and their source.

Variables	Type of variable	Values	Data source
Presence/Absence of mugger crocodile	Binomial (Dependent variable)	1 = Presence; 0 = Absence	Field survey
Water Depth (WD) (m)	Continuous	Range (1.7–10)	Field survey
Slope (°)	Continuous	Range (2–12)	Field survey
Elevation (m)	Continuous	Range (80–98)	Field survey
Distance to Road (DR) (m)	Continuous	Range (14–2671)	(OCHA Nepal, 2021a)
Distance to Settlement (DS) (m)	Continuous	Range (98–4962)	(OCHA Nepal, 2021b)
Bank Substrate (BS)	Categorical	1 = Grassy bank, 2 = Muddy and grassy bank, 3 = Muddy bank, 4 = Sandy and grassy bank, 5 = Sandy and muddy bank, 6 = Sandy bank	Field survey
Human disturbances (HD)	Categorical	1 = Yes, 0 = No	Field survey

### 2.3 Data analysis

**Figure 2:**
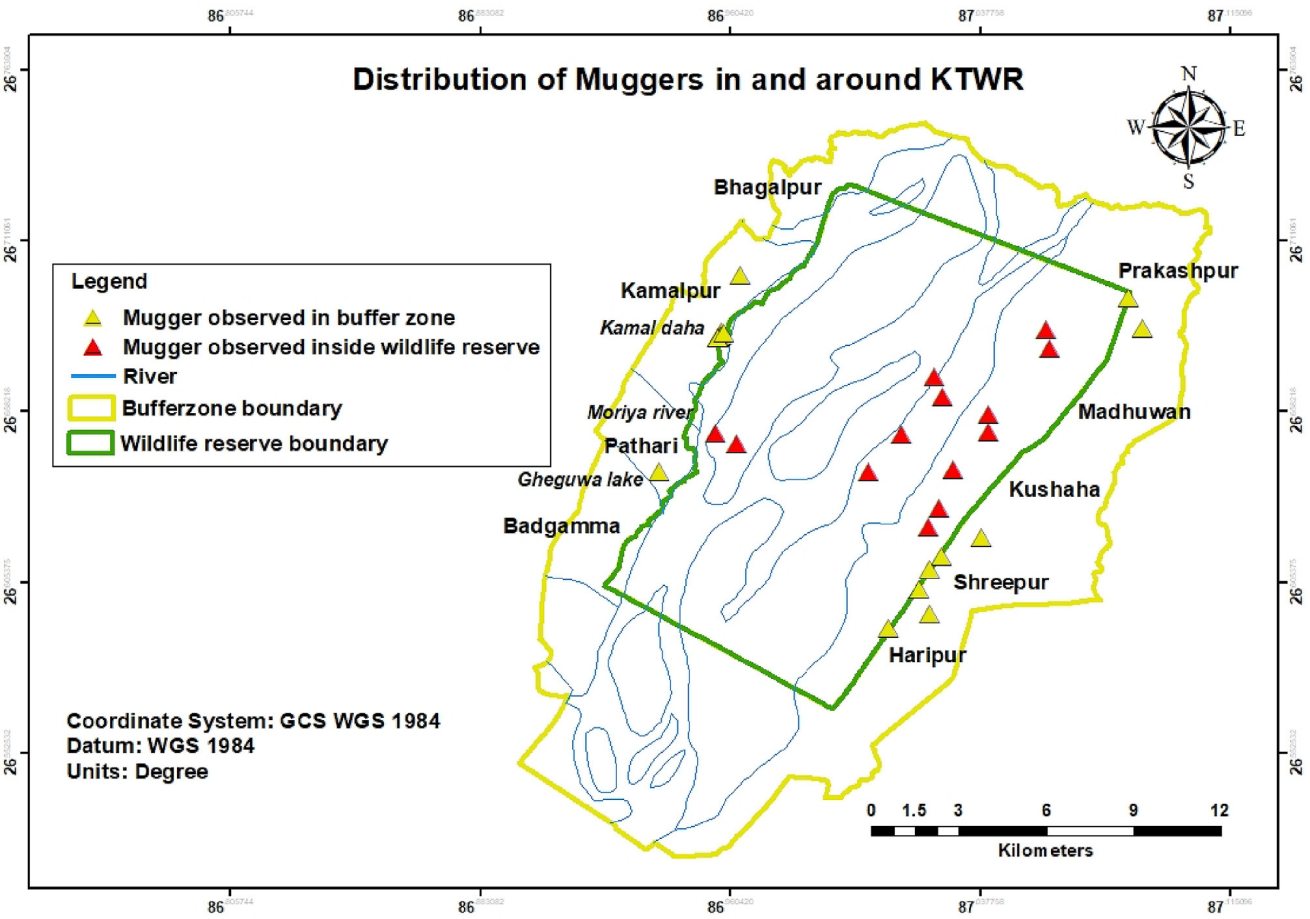
Distribution of muggers in and around KTWR.

The numbers of the muggers recorded during the population survey were presented in a tabulated format according to the age size class (Table 3). The distribution map of the muggers in an around the KTWR was prepared in ArcGIS 10.5 (ESRI, 2017) (Figure 2).

**Table 3:** Details of the mugger sighted.

S.N	Transects	Site characteristics	Total mugger sighted	Juvenile	Sub-adult	Adult
1.	I	River	6	2	2	2
2.	II	Pond and river	8	4	2	2
3.	III	Chain of private ponds	10	5	3	2 (1∗)
4.	IV	Community Ponds, river	10	1	5	4
5.	V	River	1	–	–	1
	Total		35	12	12	11

Note: All the muggers were basking. ∗ = swimming.

Presence/absence of the mugger was considered as dependent variables in the analysis. Multicollinearity test was performed for all the selected independent variables based on Variance Inflation Factor (VIF) using the Package ‘Faraway’ (Boomsma, 2014) in R 4.0.4 (R Core Team, 2021). All the seven variables were selected for the final analysis as they did not show any multicollinearity with having a tolerance value more than 0.1 and VIF less that 10 (Bowerman and O’connell, 1990). After that, we used generalized linear models (GLMs) under binary logistic regression to quantify the probability of sighting muggers with respect to different habitat predictors. Out of seven selected predictors, we used bank substrate and human disturbance as a categorical variable and remaining other as a continuous variable. For running the analysis, we used two packages ‘Desctools’ (Signorell et al., 2018) and ‘Manipulate’ (Racine, 2012) in R 4.0.4. Similarly, for testing the significance level, Wald Z test (Johnson, 1998; Macnally, 2000) was applied.

Furthermore, we used the ‘dredge’ function under the package ‘MuMIn’ (Barton, 2009) in R version 4.0.4. All possible models were constructed and they were ranked based on small-sampled AICc. Models with the lowest AICc indicate the best or dominant model (Barton and Barton, 2020). The final models were obtained by averaging the top candidate models (delta AIC ≤2) (Burnham and Anderson, 2001). Also, we generated ROC curve and AUC value to test predictive performance of the selected model using the package ‘ROCR’ (Sing et al., 2005) (see Figure 4).

## 3 Results

### 3.1 Population status and distribution of mugger crocodile in and around KTWR

**Figure 3:**
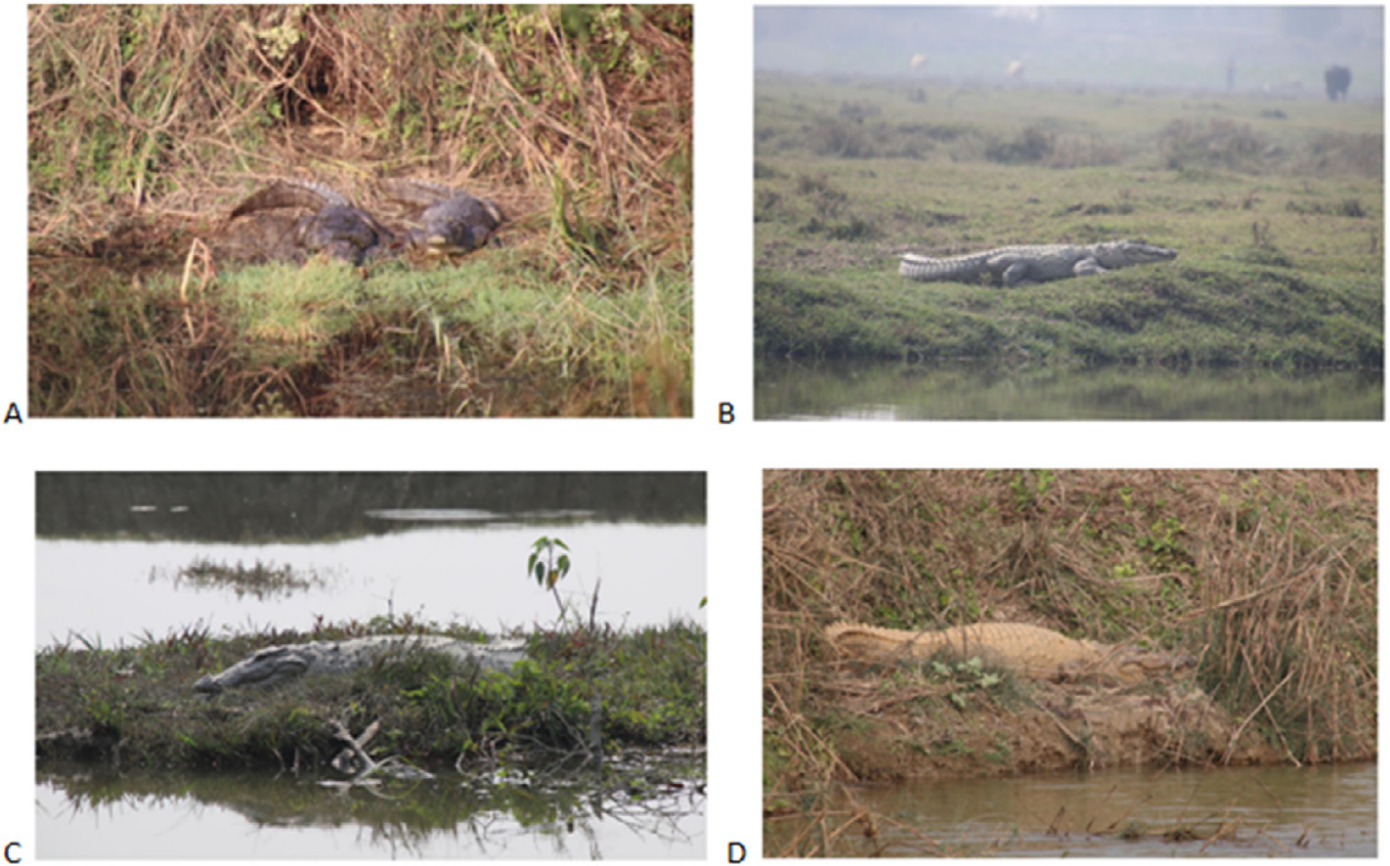
Mugger observed in and around KTWR A) mugger’s basking in the Gohi Tal B) mugger basking in the Kamal Daha pond C) mugger basking in a mud island in Kamal daha pond D) mugger basking in the Moriya river (tributary of Koshi river).

During the survey, in total we recorded 35 individuals of muggers in and around Koshi Tappu Wildlife Reserve as a proxy of minimum population size (Figure 3). Of the total 35 muggers, 22 individuals were sighted in ponds (private ponds, community ponds and ponds inside reserve) while 13 were sighted in the Koshi river, branches of the Koshi river and Moriya river. Sixteen muggers were sighted inside the reserve and remaining 19 muggers were sighted in the buffer-zone area. The total length of the muggers was visually estimated within age-size categories: juveniles (N = 12); sub-adults (N = 12), and adults (N = 11). However, hatchlings and yearlings were not sighted during the study period. Out of the total mugger sighted; 31 individuals were observed basking, an individual was swimming and three of them were displaying mouth gaping behavior. The details of the muggers sighted are presented in (Table 3).

**Figure 4:**
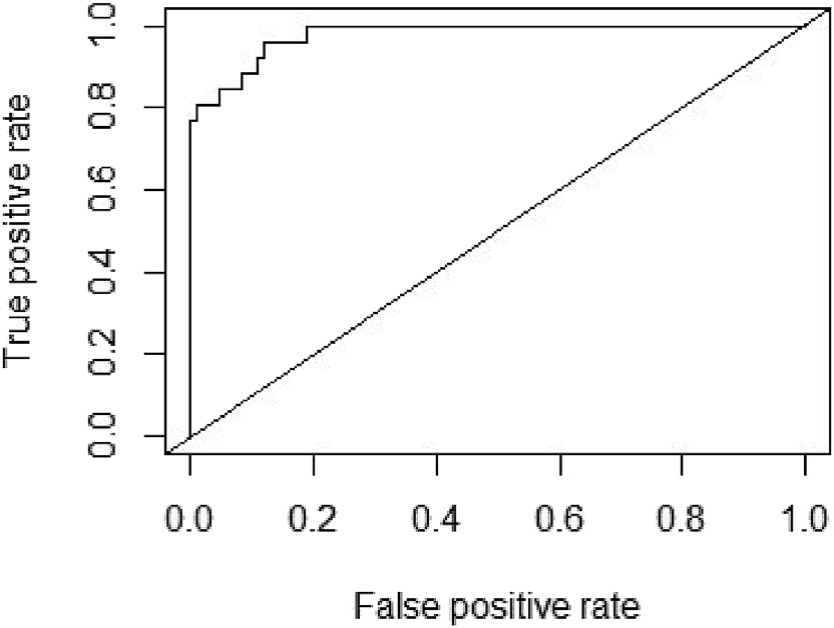
ROC curve for the model with binomial structure (GLM with binary logistic regression).

### 3.2 Mugger’s occurrence probability in relation to different habitat predictors

Out of 110 sampling stations, we recorded muggers from 26 stations. The highest number of muggers observed from a single station was 4. We observed single mugger from 21 stations. We did full model averaging to compute the effect of assigned habitat predictors. Out of seven habitat predictors examined in the study, water depth (b = 1.780, s. e. = 22.14, p = 0.003) and slope (b = 0.865, s. e. = 0.33, p = 0.01) affected the probability of observing muggers significantly in and around KTWR. Results of the model demonstrated that probability of observing mugger increased with the increase in water depth and slope. However, there were no significant differences in the probability of observing muggers for other three continuous variables: elevation, distance to roads, and distance to settlements (Table 4).

**Table 4:** Predictors associated with probability of Muggers occurrence in KTWR.

S. N.	Predictors	Estimate	Std. Error	z value	Pr (>|z|)
1.	(Intercept)	17.27	22.14	0.78	0.43
2.	WD	1.78	0.60	2.95	0.003∗∗
3.	Slope	0.865	0.339	2.549	0.010∗
4.	Elevation	−0.33	0.26	−1.26	0.20
5.	DR	−0.0009	0.0007	−1.24	0.21
6.	DS	−0.0002	0.0005	−0.57	0.56
7.	factor(BS)2	0.37	1.59	0.23	0.81
8.	factor(BS)3	−0.59	1.92	−0.30	0.75
9.	factor(BS)4	−4.30	3.33	−1.28	0.19
10.	factor(BS)5	0.003	1.93	0.002	0.99
11.	factor(BS)6	1.88	2.32	0.81	0.41
12.	factor(HD)1	0.88	1.48	0.60	0.54

Signif. codes: 0 ‘∗∗∗’ 0.001 ‘∗∗’ 0.01 ‘∗’ 0.05 ‘.’ 0.1 ‘ ’ 1.

Where, WD = Water Depth, DR = Distance to river, DS = Distance to Settlement, BS = Bank substrate.

For the analysis, all other bank substrate types (coded 2 to 6) were compared with grassy bank (coded 1). Similarly, for the human disturbance types, the presence of human disturbance in each station “Yes” (coded 1) was compared with absence of human disturbance in each station “No” (coded 0) in the analysis. Nevertheless, both the categorical variables were also insignificant to probability of observing muggers in and around KTWR. The detail of the mugger’s occurrence among two categorical habitat predictors is illustrated in the (Table 5).

**Table 5:** Details of mugger’s presence along two categorical predictors (bank substrate and human disturbance).

Predictors	Factors	Total number of sampling points	Number of stations with muggers observed	Proportion observed at each sampling point
Bank substrate	Grassy bank	12	5	0.42
	Muddy and grassy bank	29	7	0.24
	Muddy bank	14	1	0.07
	Sandy and grassy bank	25	2	0.08
	Sandy and muddy bank	22	10	0.4
	Sandy bank	8	1	0.13
Human disturbance	Yes	74	20	0.27
	No	36	6	0.17

The model with distance to road, elevation, slope and water depth appeared as best model among the predictors sets (Table 6). However, similarity in model weights creates model uncertainty among the assigned habitat predictors.

**Table 6:** Second-order Akaike Information criterion scores (AICc, ΔAIC & AIC weight) of a generalized linear model with binomial structure predicting the factors responsible for the muggers observation.

Component models	df	AICc	ΔAIC	Weight	loglike
Binomial distribution					
DR + Elevation + Slope + WD	5	50.5	0.00	0.176	−19.966
DR + Slope + WD	4	50.7	0.15	0.163	−21.139
DR + HD + Slope + WD	5	51.4	0.94	0.110	−20.433
DR + Elevation + HD + Slope + WD	6	51.6	1.09	0.102	−19.390

The area under Receiver operating curves (ROC) for the dominant model (GLM with binary logistic regression) were estimated to be 0.97 with an accuracy value of 0.9 (94.54 %) (Figure 4).

## 4 Discussion

The study highlights the current population status and habitat use by mugger crocodiles in and around KTWR. The survey recorded a total of 35 muggers, which evidently has increased compared to the findings of Goit and Basnet (2011) and Baral and Shah (2013) who recorded 21 and 16 individual muggers respectively. The current increment in population data of muggers in and around KTWR is likely due to extensive survey in a larger geographical area including the natural, private and community ponds of the adjoining buffer zone area. The KTWR and other conservation agencies have supported local communities for fish farming in private and community fishponds (Bhattarai et al., 2021). The fish stock in community and private fishponds for commercial farming has become easy source of prey for muggers and pressurized disturbance in the river systems might have effectuated the local migration of muggers from river systems to such ponds. This local migration of mugger crocodiles in community and private fishponds is likely to increase human-mugger crocodile conflict in future. However, Bhattarai et al. (2021) reported that local communities of Kamal daha area have higher tolerance level in community managed fishponds and they also worship muggers in and around KTWR. Majority of the muggers were observed basking during the survey. In winter season, crocodiles spend most of the time basking during daytime (Acharya et al., 2017). Active animals commonly seek to maintain body temperatures of 30–33 °C through behaviors such as basking (Grigg and Gans, 1993).

Burbidge (1987) and Webb (1991) mentions that low to moderate water current is a characteristic of crocodile’s habitat, and they tend to avoid rapid and turbulent water. We found similar results in our study as a few numbers of muggers were observed in the main Koshi river which has high water current. The observed daily flow data (from 1977 to 2008) at the outlet station of Koshi river basin showed that it carries a flow of 1500 m^3^ s^−1^ on average (DHM, 2008; Devkota and Gyawali, 2015). However, the inter-annual variation ranges from 618 m^3^/s to 2055 m^3^/s. Most muggers were observed in ponds with stagnant water and oxbow lakes created by Koshi river. Also, such oxbow lakes had piled logs and grasses which likely served as shelter The mugger is an opportunistic predator, and it can also use piled logs, roots, debris for ambush and also stores its food under such logs, roots and debris for later use (Bhattarai, 2015). However, during our survey, we did not observe any mugger storing food or attempting to hide; but upon disturbance some basking individual mugger retreated into water. In addition, the distribution of the muggers was higher in the boundary of the KTWR than the core areas in both eastern and western side of the KTWR. This is likely due to higher food competition of muggers with licensed fishermen in the KTWR and unregulated disturbance. Between 17th September, 2020 and 15th December, 2020, the KTWR has provided 466 fishing licenses to indigenous fishing community (KTWR pers. comm). The plausible competition for food in the KTWR with the fishermen and easy prey availability in the private pond might have attributed the muggers' shift to the boundary and ponds of the buffer zone area.

In our study, the slope of the stations ranged from 2 to 12° with minimal fluctuations. The study conducted by Lamichhane et al. (2021) found that the probability of sighting mugger did not significantly differ with slope. In contrast, in this research, slope was a significant predictor in the probability of observing mugger, which increased in steeper slopes. Similar results were observed in the study conducted by Choudhary et al. (2018) on the muggers of Katarniaghat Reserve, India. In contrast, Neupane et al. (2020) observed higher probability of occurrence of Gharials in gentler slope. The preference for shallower slopes and lower height by Gharial is because it is difficult for them to negotiate places that are steep and elevated, owing to their weak legs (Bustard and Singh, 1978; Whitaker and Basu, 1983). Gharial can only crawl on land, unlike mugger, which have a high walk (Choudhary et al., 2018). Hussain (2009) suggests water depth as important gradient for Gharials, as it allows them to avoid threats from disturbance by retreating to deep water immediately for safety purposes. We observed similar results on the muggers of KTWR as probability of occurrence of the muggers was significant with respect to water depth.

The predictors such as human disturbance, bank substrate, distance to road, distance to settlements and elevations were found to be insignificant. More number of muggers was observed in the private fish ponds in the buffer zone area, possibly due to easy prey availability in the fish ponds. Venugopal and Deviprasad (2003) in their study in Raganthittu Bird Sanctuary also mentioned less wariness in the mugger crocodiles in regular tourist boat zones towards presence of human and boats. The observations of Dave and Bhatt (2021) on basking behavior of mugger crocodile in Pond Deva noted few anthropogenic activities, like washing cloth or grazing cattle in the shores had a little effect on the basking species. The muggers in and around KTWR possibly might have been acclimatized to the human presence, pertaining to persistent interchange with fishermen and local people of the area. This might be one of the reason of occurrence of sighting mugger did not differ significantly with respect to human disturbance, distance to settlement and distance to road. Choudhary et al. (2018) states that muggers can bask on a variety of basking substrate, which includes sand, silt, rock, and fallen logs. We obtained consistent results, as probability of sighting muggers in the KTWR did not significantly differ with respect to bank substrate. Lamichhane et al. (2021) also depicts bank substrate as insignificant factor in probability of observing mugger at Ghodaghodi Lake Complex, Nepal. According to Fukuda et al. (2007) elevation range of certain catchment areas was considered as appropriate measure for the crocodile’s habitat, as each segment of a catchment has its area at sea level. The elevation domain of the stations of the study area ranged from 80 to 98 (m) elevation. This infinitesimal difference in the elevation range in the study area might be the explanation, why the probability of occurrence of mugger did not significantly differ with respect to elevation.

## 5 Conclusion

The study recorded total of 35 muggers in and around KTWR which is the highest number documented so far. In comparison to the reserve, more muggers were seen in the buffer zone locations. In ponds with stagnant water and seeps created by the slowly moving Koshi River, more muggers were observed. The probability of observing muggers was found significant with respect to slope and water depth in the study area. The odds of observing muggers increased with the increase in water depth and slope range. The likelihood of witnessing muggers was found to be unaffected by habitat variables such bank substrates, human disturbance, distance to road, distance to river, and elevation. Muggers were more prevalent in the buffer zone area and private fish ponds, posing a threat to both fish farmers and the muggers themselves. This scenario emerges with the plausibility of generation of human mugger conflict in the near future. In order to stifle the muggers inside the reserve, the reserve authority shall play a crucial role through proper habitat management and by regulation of the fishing activities inside the KTWR.

## Declarations

### Author contribution statement

Divya Bhattarai, Saurav Lamichhane: Conceived and designed the experiments; Performed the experiments; Analyzed and interpreted the data; Contributed reagents, materials, analysis tools or data; Wrote the paper.

Pratik Pandeya: Performed the experiments; Wrote the paper.

Santosh Bhattarai: Conceived and designed the experiments; Contributed reagents, materials, analysis tools or data; Wrote the paper.

Jeetendra Gautam: Conceived and designed the experiments.

Ram Chandra Kandel: Contributed reagents, materials, analysis tools or data.

Chiranjibi Prasad Pokheral: Conceived and designed the experiments; Contributed reagents, materials, analysis tools or data.

### Funding statement

This work was supported by the Crocodile Specialist Group, Australia and 10.13039/100010227British Herpetological Society, UK.

### Data availability statement

Data will be made available on request.

### Declaration of interests statement

The authors declare no conflict of interest.

### Additional information

No additional information is available for this paper.
